# Fundamental Characteristics of AAA+ Protein Family Structure and Function

**DOI:** 10.1155/2016/9294307

**Published:** 2016-09-14

**Authors:** Justin M. Miller, Eric J. Enemark

**Affiliations:** Department of Structural Biology, St. Jude Children's Research Hospital, 262 Danny Thomas Place, Memphis, TN 38105, USA

## Abstract

Many complex cellular events depend on multiprotein complexes known as molecular machines to efficiently couple the energy derived from adenosine triphosphate hydrolysis to the generation of mechanical force. Members of the AAA+ ATPase superfamily (ATPases Associated with various cellular Activities) are critical components of many molecular machines. AAA+ proteins are defined by conserved modules that precisely position the active site elements of two adjacent subunits to catalyze ATP hydrolysis. In many cases, AAA+ proteins form a ring structure that translocates a polymeric substrate through the central channel using specialized loops that project into the central channel. We discuss the major features of AAA+ protein structure and function with an emphasis on pivotal aspects elucidated with archaeal proteins.

## 1. Molecular Machines Are Ubiquitous in the Cell

All cells use sophisticated protein complexes that couple the chemical energy of nucleoside triphosphate (NTP) hydrolysis to the generation of mechanical force [1]. These complexes operate analogously to an engine, with NTP as the fuel that is combusted to overcome thermodynamic barriers. Based on this similarity, these protein complexes are termed “molecular machines” [2]. A diverse set of molecular machines are required for a myriad of cellular functions including DNA replication [3, 4] and recombination [5], regulated proteolysis [6–8], protein disaggregation [9–12], protein complex disassembly [13], and many others.

The AAA+ superfamily of ATPases (ATPases Associated with various cellular Activities) are critical parts of many molecular machines [14]. AAA+ proteins catalyze the hydrolysis of adenosine triphosphate (ATP) and use the derived energy to perform mechanical work. The AAA+ domain architecture and the underlying ATP hydrolysis mechanism are highly conserved. Members of this family often function as oligomers with ATPase sites at the interfaces of adjacent subunits. Both subunits contribute residues to a bipartite ATPase active site such that these catalytic features are either* cis*- or* trans*-acting. These proteins participate in many diverse cellular events, assisted by additional modules appended to or inserted in the AAA+ domain. Archaeal AAA+ proteins have allowed the elucidation of several critical features of AAA+ structure, function, and mechanism. For example, the crystal structure of* Pyrobaculum aerophilum* Cdc6 provides an extremely high resolution view for how a AAA+ protein binds nucleotide, its associated magnesium ion, and the water molecules that complete the magnesium octahedral coordination sphere [15]. Here we highlight distinguishing structural and functional features of AAA+ proteins, highlighting critical features of AAA+ structure and function elucidated with archaeal proteins.

## 2. Secondary and Tertiary Structural Features Defining the AAA+ Protein Fold

**Figure 1:**
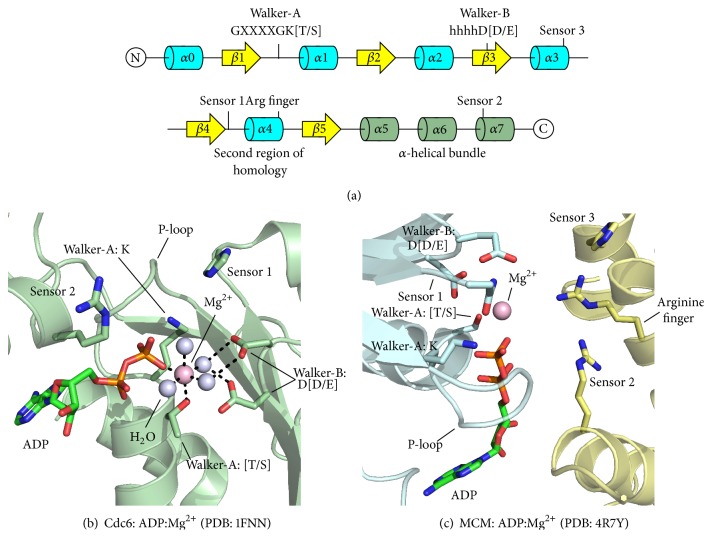
Features of the AAA+ ATPase domain. (a) The AAA+ *α*-*β*-*α* fold topology and active site feature locations are shown in primary sequence and secondary structure. Helices and strands within the core *α*/*β* fold are colored in blue and yellow, respectively. C-terminal lid domain helices are colored in light green. (b) Active site residues are precisely positioned to bind nucleotide and Mg^2+^. The Mg^2+^ cation is directly coordinated by the Walker-A threonine, the *β*-phosphate of the bound nucleotide, and four water molecules. Dashed lines indicate discussed molecular interactions (see text). The bound ADP molecule and critical active site features are shown in stick, water molecules as light blue spheres, and the magnesium ion as a magenta sphere. (c) The ATPase site forms at subunit interfaces by residues of adjacent subunits. The Walker-A, Walker-B, and Sensor 1 residues are positioned on the left side of the site and all reside on the same subunit (blue, “*cis*-acting”), while three basic residues are located on the right side of the site from the neighboring subunit (yellow, “*trans*-acting”). Bound ADP and Mg^2+^ are represented identical to (b). The protein topology cartoon in (a) was prepared using the TopDraw software package [42]. All structure representations in the figure were prepared with the Pymol software package [43] and PDB accession codes 1FNN [15] (b) and 4R7Y [41] (c).

The AAA+ family is a subset of the larger P-loop protein superfamily [16]. All P-loop family members are structurally similar and possess a distinct *α*/*β* fold. The AAA+ domain contains 200–250 amino acids with a central *β*-sheet in *β*5-*β*1-*β*4-*β*3-*β*2 strand order (Figure 1(a)). The *β*-sheet is flanked on both sides by *α*-helices to form a three-tiered *α*-*β*-*α* sandwich. Features that distinguish AAA+ family members from other P-loop NTPases include the insertion of *β*4 between *β*1 and *β*3 [17], the lack of an antiparallel *β*-strand adjacent to *β*5 [18], and the lack of any additional strands directly adjacent to either *β*5 or *β*2 [16–18]. This contrasts other P-loop family members that contain additional *β*-strands, such as the ABC subfamily [18, 19].

Many AAA+ proteins have a C-terminal *α*-helical bundle in addition to the *α*-*β*-*α* core (Figure 1(a)). The functional roles of the helical bundle are varied and include the formation of a lid over the nucleotide binding site and mediation of subunit interactions in oligomeric protein complexes. The position of the helical bundle relative to the *α*-*β*-*α* core fold is often nucleotide-dependent. For example, the C-terminal bundle of the HslU protein translocase rotates 21.5° between the fully open state (apo state) and closed state (ADP-bound) with the ATP-state representing an intermediate conformation [20].

## 3. Distinguishing AAA+ Primary Sequence Motifs

The AAA+ domain contains primary sequence motifs originally used to establish the AAA family [14, 16, 17, 21]. The AAA+ protein family was defined after the crystal structures of N-ethylmaleimide-sensitive fusion (NSF) protein and the *δ*′ subunit of the* E. coli* DNA polymerase III were considered alongside multiple sequence alignments and revealed a unique region C-terminal to *β*4 [14, 22, 23]. We will discuss here the signature AAA+ sequence motifs and their functional roles.

### 3.1. Walker-A and Walker-B Motifs

Like all P-loop NTPases, AAA+ proteins have Walker-A and Walker-B motif residues that are critical for binding and hydrolyzing ATP. The Walker-A motif consists of a GXXXXGK[T/S] sequence, where X is any amino acid and the C-terminal residue is either threonine or serine. Structurally, this motif forms a loop between *β*1 and *α*1 within the AAA+ topology (Figures 1(a) and 1(b)) [16, 17, 21]. This feature is the canonical P-loop and is one of the most strongly conserved sequences for AAA+ proteins. Minor deviations include NtrC (GXXXXGK[D/E]), MCM (GXXXXGAKS), and MoxR (GXXXXAK[T/S]) [16].

The Walker-B motif spans *β*3 and is characterized by the sequence, hhhhD[D/E], where h represents any hydrophobic residue and the C-terminal residue is aspartate or glutamate (Figures 1(a) and 1(b)). ATP hydrolysis catalyzed by AAA+ proteins depends on the Walker-B glutamate residue at the C-terminus of *β*3 (Figure 1(b)) [16]. This conserved glutamate residue is the catalytic base that activates a water molecule for nucleophilic attack on the *γ*-phosphate during ATP hydrolysis (Figure 1(b)) [16, 17, 24, 25]. Mutation of the Walker-B glutamate therefore prevents ATP hydrolysis but still allows ATP binding [26–29]. For this reason, the Walker-B glutamate can be mutated to glutamine or alanine to dissect the roles of ATP binding and ATP hydrolysis in AAA+ protein activities [17, 26, 29, 30].

The X-ray crystal structure of* Pyrobaculum aerophilum* Cdc6 bound to ADP and Mg^2+^ shows the atomic roles of the Walker-A and Walker-B motif residues in binding ATP [15]. The conserved Walker-A lysine residue at the start of *α*1 (Figure 1(a)) directly interacts with the phosphate groups of the bound nucleotide (Figure 1(b)). The Walker-A lysine is commonly mutated to alanine [31] to globally disrupt nucleotide binding since this residue is so intimately involved in nucleotide binding. A Mg^2+^ cation has an octahedral coordination geometry that consists of the Walker-A threonine, the *β*-phosphate of the bound nucleotide, and four water molecules (Figure 1(b)). Some of Mg^2+^-coordinating water molecules interact directly with the Walker-B acidic residues (Figure 1(b)). Other than water-mediated interactions, the Walker-B residues do not directly interact with ATP or magnesium (Figure 1(b)) [15].

### 3.2. Second Region of Homology

All AAA+ proteins contain a region C-terminal to the Walker-B motif termed the Second Region of Homology (SRH). This region spans 15–20 residues to include part of *β*4, the entire *α*4 helix, and the loop connecting *α*4 to *β*5 (Figure 1(a)) [17, 32]. The SRH contains the Sensor 1 and Arginine finger motifs, both of which are required for ATP hydrolysis. These features coordinate nucleotide hydrolysis and propagate conformational changes associated with nucleotide hydrolysis between subunits in AAA+ protein complexes [17]. Due to the functional importance and the lack of SRH in other Walker-type NTPases, this region serves as a defining characteristic of the AAA+ family.

The Sensor 1 motif is located at the N-terminal end of the SRH in the loop connecting *β*4 to *α*4 (Figure 1(a)). Sensor 1 is a polar residue that is most commonly asparagine but can also be serine, threonine, or aspartate [14, 19]. It is structurally located between the Walker-A and Walker-B motifs and functions in concert with the Walker-B glutamate to correctly orient the nucleophilic water molecule that undergoes attack on the *γ*-phosphate of the bound ATP molecule [19]. As such, Sensor 1 is critical for proper ATPase function as well as any function that may be coupled to ATP hydrolysis. For example, mutation of the Sensor 1 asparagine in the ATP-dependent protease FtsH results in a loss of protease activity even though this motif is not located in the domain that performs proteolysis [33].

The AAA+ ATPase site is at the interface of adjacent subunits in a protein complex (Figure 1(c)). The Walker-A/B and Sensor 1 residues of the ATPase site are all located on the same subunit, while the arginine finger is derived from the neighboring subunit. For this reason, the Walker-A/B and Sensor 1 residues are defined as “*cis*-acting” residues while the arginine finger is “*trans*-acting.” In primary sequence, the arginine finger is located near the C-terminal end of the SRH and is located in the loop between *α*4 and *β*5 [17]. This residue is nearly always an arginine, though occasionally a lysine residue is present [19]. The name “arginine finger” is derived from a structural similarity with GTPase activator proteins such as the Ras-RasGAP complex, where an arginine residue is observed in crystal structures directed into the GTP binding site via a “finger loop” [34]. For all known structures of AAA+ oligomers, a similar arginine residue projects into the ATP binding and hydrolysis site of an adjacent subunit. Based on the GTPase activator proteins, the arginine finger forms intermolecular interactions with the *γ*-phosphate of the bound nucleotide that may stabilize an accumulated negative charge that occurs in the transition state during hydrolysis [32, 35]. Mutational studies with FtsH and NtrC conclude that the arginine finger is necessary for hydrolysis, but not ATP binding [33, 36]. Mutation of the arginine finger in* Sulfolobus solfataricus* MCM [37],* Escherichia coli* RuvB [38], and others revealed similar results with an ablation of observable ATPase activity. Mutation of the arginine finger to glutamate in HslU not only impairs ATP hydrolysis but also disrupts oligomerization [32, 39].

### 3.3. Sensor 2 and 3 Residues

Two additional active site features have been identified from examination of AAA+ protein sequences and structures. The first is the Sensor 2 feature, which mediates conformational changes associated with a cycle of ATP binding and hydrolysis [32]. This generally occurs through direct interaction of a Sensor 2 residue with the *α*-phosphate of the bound ATP molecule, which is consistent with reports that mutations at this position diminish nucleotide binding [17, 32, 35, 40]. Sensor 2 is conserved in all AAA+ proteins as an arginine or lysine and is located near the beginning of *α*7. AAA family members, as opposed to AAA+ family members, generally have an alanine residue in the Sensor 2 position [16]. Sensor 2 functions as a* cis*-acting residue in AAA+ proteins that contain C-terminal lid domains and is a* trans*-acting residue in proteins lacking a canonical *α*-helical lid domain. One example of a* trans*-acting Sensor 2 is in MCM proteins, where an insertion in the C-terminal *α*-helical bundle disrupts the canonical lid domain and positions the Sensor 2 arginine as a* trans*-acting residue (Figure 1(c) shows Sensor 2 from an archaeal MCM) [41]. Similarly, papillomavirus E1 lacks a canonical lid domain and contains a* trans*-acting lysine residue in the Sensor 2 structural position [40].

An additional sensor residue, termed Sensor 3, is present in the structures of the AAA+ hexameric helicases papillomavirus E1 [40] and MCM proteins [41, 51]. To date, this motif has been observed as either an arginine or a histidine in E1 or MCM, respectively (Figure 1(c)). This residue may have a role in stabilizing the ATP-state, where the E1 structure reveals a* trans*-acting arginine that reaches across the subunit interface to interact with a Walker-B aspartate and Sensor 1 asparagine [40]. The 3.8 Å cryo-EM structure of the eukaryotic Mcm2–7 helicase shows that the Sensor 3 histidine is similarly positioned to the arginine of E1, and it interacts with the Walker-B glutamate when at the tightest interfaces [51]. This residue may have a role in stabilizing the compact subunit interface that is needed for the ATP-state.

### 3.4. N-Linker

There are defining features of the AAA+ domain N-terminal to *α*0 [16, 17]. This region typically contains a conserved glycine or a similarly small residue that forms a cap at the N-terminus followed by another family-conserved residue [16, 17, 52]. Based on multiple sequence alignments, Smith and coworkers have classified proteins in groups based on the residue after the first glycine, where PRS- and p97-like proteins have another glycine, and HslU and the Clp ATPases have a hydrophobic residue [52]. There is a conserved region N-terminal to this dipeptide sequence that runs perpendicular to the *β*-strands of the *α*-*β*-*α* core [16]. This region is referred to as the “N-linker” and both contributes to the ATP binding pocket and serves to connect the AAA+ domain to other domains within a protein [52]. Due to the positioning of the N-linker between domains of a protein and directly adjacent to the ATP binding site, this motif may play a direct role in coupling ATP hydrolysis to conformational changes. For example, the isoleucine-glycine N-linker of HslU interacts with the nucleotide adenine ring [20, 49, 52]. Comparison of nucleotide-bound and nucleotide-free HslU structures reveals a repositioning of the isoleucine side-chain upon nucleotide binding such that the side-chain is excluded from the ATP binding pocket and the glycine residue changes conformer.

## 4. Structural Features Define the AAA+ Clades

Though all AAA+ family members contain common features that include the Walker motifs, the Second Region of Homology, and so forth, many of these proteins also contain insertions of specific secondary structural elements within or near the core *α*-*β*-*α* fold. As a result, the AAA+ family members have been classified in subdivisions based on specific sequence and structural properties [16, 19]. These subdivisions have been thoroughly reviewed previously [16, 19], and so we will not discuss these classifications in depth. A brief overview of each clade is provided below.

**Figure 2:**
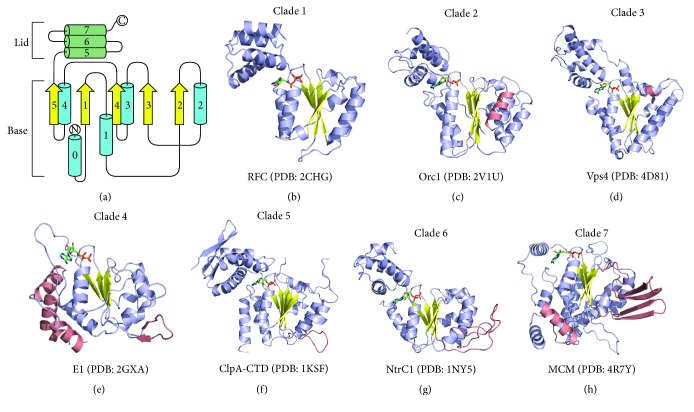
Clade-specific features of the AAA+ domain. (a) The simplest AAA+ domain is characterized by an *α*-*β*-*α* topology with a C-terminal *α*-helical lid domain. Helices and strands within the base domain are shown as blue cylinders and yellow arrows, respectively, and lid domain helices are represented by green cylinders. (b–h) A crystal structure of a characteristic member of each clade is shown in cartoon representation with helices and strands colored as blue and yellow, respectively. The insertions that distinguish each clade are highlighted in salmon. Each structure contains a bound nucleotide molecule that is shown in stick. The protein topology cartoon in (a) was prepared using the TopDraw software package [42]. All structure representations in the figure were prepared with the Pymol software package [43] and PDB accession codes 2CHG [44] (b), 2V1U [45] (c), 4D81 [46] (d), 2GXA [40] (e), 1KSF [47] (f), 1NY5 [48] (g), and 4R7Y [41] (h).

*Clade 1: Clamp Loader Clade*. The clamp loader clade represents the minimal AAA+ domain without any modifications (Figures 2(a) and 2(b)) [16, 19]. As shown in Figure 2(a), this includes the previously discussed *α*-*β*-*α* sandwich with strand order *β*5-*β*1-*β*4-*β*3-*β*2 and a C-terminal *α*-helical lid domain. Clamp loaders are conserved in bacteria, archaea, and eukaryotes and are required to load the ring-shaped processivity clamps that maintain continuous association between DNA polymerases and replicating DNA [53, 54]. This family includes Replication Factor C (RFC), *γ*/*δ* DNA polymerase III subunits, and WHIP families, where each family possesses unique structural features outside of the core AAA+ protein fold [44, 55, 56]. Examples include a unique Zn cluster insertion downstream of the Walker-A motif in bacterial *γ*/*δ* DNA polymerase III subunits [22] and a distinct C-terminal globular domain fused to the AAA+ domain in the WHIP family [16].

*Clade 2: Initiator Clade*. Members of the initiator clade include all cellular origin recognition proteins and helicase-loading proteins from bacteria, archaea, and eukaryotes [19]. Clade 2 is characterized by the insertion of an extra *α*-helix between *α*2 and *β*2 within the *α*-*β*-*α* core (shown in salmon in Figure 2(c)). The two major families within Clade 2 are the DnaA/DnaC and Orc/Cdc6 groupings, which are derived from bacteria or archaea/eukaryotes, respectively. Archaeal initiators and DnaA share a common domain organization that includes an initiator-type AAA+ domain with a C-terminal double-stranded DNA (dsDNA) binding domain [15, 45, 57–60]. Cocrystal structures of the Orc1 DNA binding domain or the Orc1 AAA+ domain bound to dsDNA reveal that both domains can independently bind to and distort DNA [45, 59]. Binding interactions between DNA and the Orc1 AAA+ domain occur through the helical initiator-specific motif (ISM) insertion associated with Clade 2 [45, 59]. Mutations in the ISM feature significantly impair initiator binding to origin DNA. In bacteria, DnaA assembles around an origin of replication and initiates local melting of duplex DNA to enable DnaC-mediated loading of the DnaB replicative helicase. Orc and Cdc6 serve a nearly identical role in eukaryotes and archaea to DnaA and DnaC, respectively, to ultimately load the replicative MCM helicase [19, 45, 61].

*Clade 3: Classic Clade*. Clade 3 represents a family of proteins that are functionally related and form closed hexameric ring structures. A defining functional feature of this family is a shared protein remodeling function. Classic AAA+ family members are defined by a short *α*-helix insertion between *α*2 and *β*2 (shown in salmon in Figure 2(d)). This structural element forms a loop that is positioned near the axial channel of the hexameric assembly and, based on sequence conservation and mutagenesis studies, has been proposed to bind substrate. In contrast to the other AAA+ subfamilies, structure-based alignments reveal that the classic clade lacks a conserved Sensor 2 arginine residue near the beginning of *α*7 [19]. Members of Clade 3 are functionally diverse, which is a result of contributions from components outside of the ATPase core. As such, Clade 3 can be subdivided to include the FtsH-, katanin-, TIP49-, AFG1-, Proteasomal-, NSF/Cdc48/Pex-, and ClpABC-families. These families are defined by the features of domains that are N- or C-terminal to the AAA+ core. For example, the FtsH family contains an N-terminal protein-interaction domain and a C-terminal Zn-protease domain. Similarly, katanin includes an N-terminal microtubule interaction domain and a C-terminal helix that may support oligomerization [13].

### 4.1. The Pre-Sensor 1 *β*-Hairpin Superclade

The AAA+ families representing Clades 4–7 constitute the “pre-Sensor 1 *β*-hairpin” (ps1*β*h) superclade, where all members share a common *β*-hairpin insertion between *α*3 and *β*4 (Figures 2(e)–2(h)) [16, 19]. Each member of this superclade contains the canonical AAA+ features, a ps1*β*h, and additional distinguishing features. Structural and biochemical studies have shown that the ps1*β*h motif is positioned near the central channel of many protein complexes [40, 41, 47, 48]. The crystal structure of the Clade 4 papillomavirus E1 protein bound to single-stranded DNA (ssDNA) revealed a ps1*β*h that projects into the central channel and directly interacts with DNA [40]. In contrast, the ps1*β*h feature is required for interaction of RuvA with the Clade 5 RuvB protein rather than for substrate translocation [62]. Therefore, the functional role of the ps1*β*h is likely clade-dependent.

*Clade 4: Superfamily III Helicase Clade*. Members of Clade 4 are exclusively viral DNA helicases that are not found in bacteria, archaea, or eukaryotes [16]. These proteins lack a C-terminal AAA+ lid domain but contain a unique helical bundle that is formed by elements N- and C-terminal to the core *α*-*β*-*α* domain (shown in salmon in Figure 2(e)) [19]. The Sensor 2 residue in this clade is based on structural position rather than sequence analysis [40] and is a* trans*-acting residue, in contrast to the* cis*-acting Sensor 2 of clades that possess a canonical lid domain. Clade 4 family members also contain the ps1*β*h insertion between *α*3 and *β*4. Superfamily III helicases form functional hexamers that belong to the AAA+ family, in contrast to Superfamily I and II helicases that contain tandem RecA-domains and function as either monomers or dimers [63]. Examples of Clade 4 proteins include the SV40 large T-antigen helicase [64, 65], papillomavirus E1 [40, 66], and the adeno-associated virus Rep40 [67]. The X-ray crystal structure of E1 bound to ssDNA shows that a ps1*β*h lysine residue of each E1 subunit forms a salt-bridge with the DNA phosphate backbone [40, 66]. The *β*-hairpin from each subunit differs in height to form a staircase-like structure that correlates with the status of the associated ATP-site [40]. This suggests a mechanism for DNA translocation, which is expected to be common to all SF3 helicases, where ATP is sequentially hydrolyzed around the ring to drive ps1*β*h movement one staircase increment at a time.

*Clade 5: HCLR Clade*. Clade 5 is the most basic member of the ps1*β*h superclade because the associated *β*-hairpin insertion is the only feature distinguishing all HCLR family members from the clamp loader proteins of Clade 1 (shown in salmon in Figure 2(f)) [19]. The HCLR clade name is derived from the four families including HslU/ClpX, ClpABC-CTD, Lon, and RuvB. The protein translocases, HslU/ClpX, ClpABC-CTD, and Lon can be broadly grouped together based on shared function, which causes the RuvB branch migrator to be classified by itself. We favor the common classification of these proteins to reflect their shared AAA+ topology. For the protein translocases, the ps1*β*h may aid in polypeptide substrate recognition rather than active translocation. In protein translocases, the ps1*β*h is positioned away from the central hexameric channel while the loop connecting *α*2 and *β*2 has been implicated in polypeptide translocation [7, 39, 68–70]. This loop between *α*2 and *β*2 projects into the central channel and is characterized in all polypeptide translocases by the sequence X-Ar-*ϕ*-X, where X, Ar, and *ϕ* are any, aromatic, or hydrophobic residues, respectively. In contrast, the ps1*β*h insertion in RuvB mediates protein-protein interactions with RuvA. Taken together, this suggests a role other than active substrate translocation for the ps1*β*h insertion in HCLR clade members. 

*Clade 6: Helix-2-Insert Clade*. Clade 6 family members differ from other ps1*β*h superclade proteins by containing an additional *β*-hairpin insertion in *α*2 that is referred to as the helix-2-insert (h2i, shown in salmon in Figure 2(g)). This clade includes the NtrC- and McrB-subfamilies, which largely differ only in function. The NtrC group activates transcription by *σ* ^54^-bound RNA polymerases by using ATP hydrolysis to drive the polymerase complex from closed to open. Clade 6 proteins that catalyze this reaction include NtrC [36, 48] and PspF [71]. In contrast, McrB, when associated with McrC, functions as a methylation-dependent restriction endonuclease [72]. Although McrB does bind ATP, it binds GTP with greater affinity, and the assembled endonuclease complex requires GTP hydrolysis to function [72–74]. While the role of the h2i in McrBC is not yet clear, mutations to the NtrC h2i impair interaction with *σ* ^54^-bound RNA polymerases [48]. Thus, the h2i in NtrC may mediate protein-protein interactions similar to the ps1*β*h of RuvB.

*Clade 7: Pre-Sensor 2 Insert Clade*. The AAA+ domain associated with Clade 7 contains ps1*β*h and h2i insertions identical to Clade 6 but differs in an additional *α*-helical insertion after *α*5 (Shown in salmon in Figure 2(h)). This insertion is located before the Sensor 2 motif and is referred to as the pre-Sensor 2 insertion (ps-2 insertion). ps-2 insertion places the C-terminal helical bundle in a different position relative to the lid domain containing clades. In the ps-2 insert clade, the C-terminal helical bundle is positioned at the backside of the *α*-*β*-*α* core in contrast to the typical configuration where the C-terminal bundle forms a lid over the top of the *α*-*β*-*α* core. This difference affects the position of the Sensor 2 motif located in the C-terminal bundle (at the beginning of the *α*7 helix, Figure 1(a)). As a result, the Sensor 2 residue is a* trans*-acting active site residue in Clade 7. Members of the ps-2 insert clade include MCM, MoxR, YifB, and dynein.

## 5. Archaeal AAA+ Hexamers Share Common Structural and Functional Features

Many AAA+ proteins share a common protein/DNA remodeling or degradation function where the energy of ATP hydrolysis is coupled to translocation along polymeric substrates [3, 6, 7, 14, 40, 41, 75–82]. These proteins commonly form a closed ring that positions central channel loops to interact with encircled DNA or polypeptide. Central channel loop motifs include ps1*β*h and h2i features and other clade-specific loop insertions. Through repeated cycles of ATP hydrolysis, the central channel loops are expected to alternate position depending on whether ATP, ADP, or no nucleotide is bound, which drives movement of bound DNA or protein substrates. The functional diversity of AAA+ proteins is achieved by additional domains or insertions to the core AAA+ domain, either N- or C-terminal, or within the domain itself. Here we will highlight the archaeal MCM, Vps4, and PAN/Cdc48 proteins, which share mechanistic features for DNA or polypeptide translocation.

### 5.1. The MCM Complex: A Hexameric Helicase at the Replication Fork

**Figure 3:**
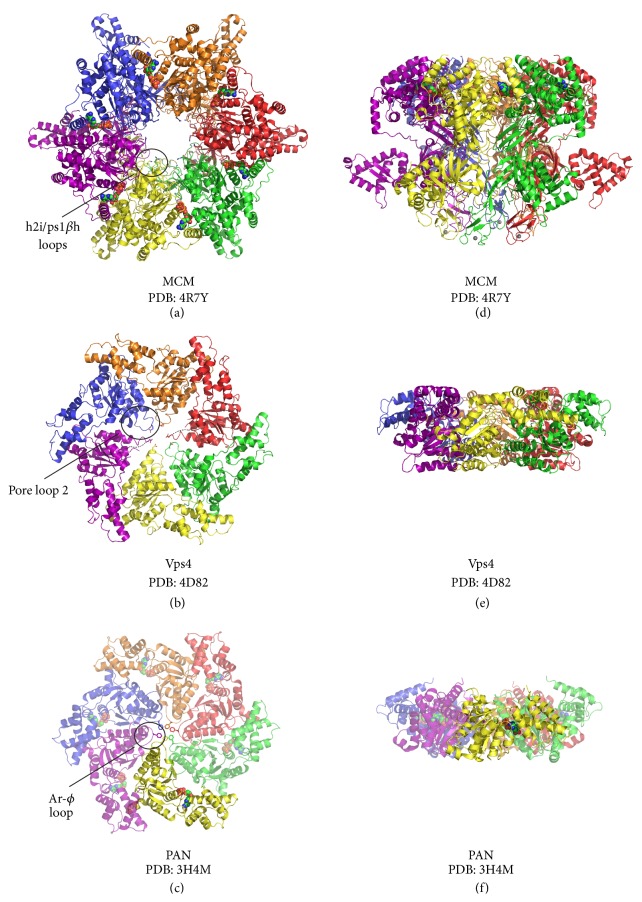
Shared structural features of archaeal AAA+ hexameric translocases. Views of MCM, Vps4, and PAN crystal structures are shown parallel (a–c) and perpendicular (d–f) to the central channel with each subunit uniquely colored and in cartoon representation. In the views parallel to the central channel, the C-terminal face of each complex is projected out of the page. In the perpendicular view, the C-terminus is located at the top and the N-terminus is located on the bottom. (c, f) The PAN hexamer is a model of the nucleotidase ring generated by superimposition of six copies of the PAN monomer atomic coordinates onto the six subunits of of the HslU hexamer (PDB: 1DO0) [49, 50]. Five of the ADP-bound PAN monomers used to generate the model of a hexamer are shown in transparent view (c, f). DNA or protein substrate interaction motifs are projected into the central channel with representative pore loops circled and labelled (a–c). Highlighted pore loop residues are shown in stick, magnesium ions as magenta spheres (a, d), ADP molecules in space-filling view (a, d, c, f), and zinc ions as gray spheres (a, d). All structure representations in the figure were prepared with the Pymol software package [43] and PDB accession codes 4R7Y [41] (a, d), 4D82 [46] (b, e), and 3H4M [50] (c, f).

The MCM complex is a hexameric ring helicase that separates parental DNA strands at the replication fork in eukaryotes and archaea. The X-ray crystal structures of archaeal MCM proteins have revealed overall architectural features that are highly conserved in both archaea and eukaryotes [3, 41, 81–85]. All MCM hexamers assemble with a two-tiered architecture with a larger C-terminal AAA+ tier and an N-terminal tier (Figures 3(a) and 3(d)) [41, 51, 86–89]. The AAA+ tier alone is sufficient to generate a DNA unwinding activity, and this activity is enhanced when the N-terminal tier is present [90, 91]. The N-terminal tier of each monomer contains an oligosaccharide/oligonucleotide binding fold (OB-fold) and a zinc binding motif. Each monomer of the C-terminal tier contains a fold that belongs to AAA+ Clade 7 (Figure 2(h)) [19]. Clade 7 proteins contain h2i and ps1*β*h features associated with Clade 6, but also a helical insertion after *α*5 that disrupts the canonical lid domain and repositions the Sensor 2 motif to be a* trans*-acting residue (Figure 1(c)). The h2i and ps1*β*h features both project into the central channel of the hexamer and contain positively charged residues that are expected to interact with DNA inside the central channel during translocation [3].

The MCM ps1*β*h and h2i features are expected to interact directly with DNA. This is similar to the E1 helicase where the ps1*β*h directly interacts with DNA in a spiral staircase-like pattern [40]. This binding mechanism implies that the central channel loops occupy different positions around the ring to form a spiral-staircase shape with loop position depending on whether ATP, ADP, or no nucleotide is bound at the associated ATP binding site. A recent cryo-EM structure for the eukaryotic helicase complex bound to DNA shows continuous electron density in the central channel that contacts h2i and ps1*β*h pore loops, suggesting direct contact between DNA and pore loops [86]. This is consistent with the structure of an archaeal MCM hexamer showing these features projected into the central channel [41]. Mutations to either the h2i or the ps1*β*h disrupt dsDNA unwinding [92, 93]. Taken together, these data suggest that h2i and ps1*β*h features directly bind DNA.

### 5.2. Vesicle Biogenesis and Endosomal Sorting Complexes: Vps4

Vps4 is a AAA+ ATPase that recycles ESCRT-III polymers (endosomal sorting complexes required for transport) from cellular membranes in both archaea and eukaryotes. Vps4 belongs to the “meiotic clade” of AAA+ ATPases, which is a subgroup of Clade 3 [13]. This group includes Vps4, katanin, fidgetin, and spastin and is characterized by an additional conserved arginine residue that directly precedes the arginine finger in the Second Region of Homology [13]. Members of this family disassemble polymeric protein substrates. Meiotic clade proteins contain an N-terminal MIT (Microtubule Interacting and Trafficking) domain and a C-terminal AAA+ domain that is distinguished by a Clade 3-specific *α*-helix insertion between *α*2 and *β*2. The loop formed between this helical insertion and *β*2 is referred to as pore loop 1 and contains a X-Ar-*ϕ*-X sequence associated with protein translocases, where X is any residue, Ar is an aromatic residue, and *ϕ* is a hydrophobic residue [46]. A second loop, pore loop 2, located between *α*3 and *β*3, contains amino acids necessary for polypeptide binding [94]. Eukaryotic Vps4 homologues also contain a unique antiparallel *β*-sheet insertion between *α*8 and *α*9 of the C-terminal helical bundle termed the *β*-domain that binds the LIP5/Vta1 activator proteins [95, 96]. The only other structurally characterized AAA+ ATPases with an insertion in this position are bacterial ClpB and ClpC, which may function as a protein substrate interaction domain [97, 98].

Vps4 is active as a hexamer and translocates polypeptide substrate using loop features that project into the central channel (see Figures 3(e)-3(f)) [13, 99]. Vps4 binds to a C-terminal helix in ESCRT-III that becomes exposed upon ESCRT-III polymerization [13, 94]. X-ray crystal structures of archaeal Vps4 proteins reveal that residues required for polypeptide binding are located on pore loops that line the interior of the central channel [99]. Loop movement is expected to proceed via a spiral staircase-like mechanism similar to the E1 helicase [3, 13, 40, 66]. Consistent with this model, biochemical studies of* Saccharomyces cerevisiae* Vps4 have clearly shown that polypeptide is bound in a ratio of one polypeptide per Vps4 hexamer and that mutations to pore loop residues disrupt polypeptide binding [94]. Further, polypeptide binding stimulates* Saccharomyces cerevisiae* Vps4 catalyzed ATP hydrolysis and stabilizes the hexameric assembly under conditions where hexamers are not observed in the absence of polypeptide substrates [94]. Taken together, the archaeal Vps4 crystal structures and the eukaryotic Vps4 biochemical data suggest that the pore loops that line the axial Vps4 channel bind to ESCRT-III polypeptide substrates, and ATP-dependent changes in loop position drive mechanical movement of ESCRT-III substrate.

### 5.3. Protein Degradation: Cdc48/PAN

ATP-dependent proteases are present in all organisms for the regulated removal of proteins involved in various cellular processes including protein signaling, heat-shock response, and cell division [6, 7]. The complexes responsible for this activity are assembled through association of a AAA+ hexamer with a compartmentalized protease to form a multitiered protein degradation machine [49, 100–105]. Through repeated cycles of ATP binding and hydrolysis, the AAA+ hexamer recognizes a degradation-tagged polypeptide substrate and then unfolds and translocates the denatured protein into the associated protease for degradation [7]. In archaea, the ATPases that catalyze this process include the Proteasome-Activating Nucleotidase (PAN) and Cdc48 [106–108]. The structures of archaeal PAN and Cdc48 are highly similar such that each protein possesses an N-terminal polypeptide substrate binding domain and a Clade 3 AAA+ domain (Figures 3(c) and 3(f)) [50, 109–111]. Each protein contains pore loop features that project into the central channel of the hexamer where PAN and Cdc48 contain pore loops 1 and 2 that are characteristic of insertions between *α*2-*β*2 and *α*3-*β*3, respectively [50, 111]. In both proteins, pore loop 1 contains the expected aromatic-hydrophobic dipeptide motif [78, 112]. Cdc48 also contains an additional pore loop N-terminal to pore loops 1 and 2 that is necessary for unfolding model substrates [113].

Cdc48 contains two tandem AAA+ ATPase domains where N-terminal and C-terminal AAA+ ATPase domains are referred to as Domain 1 (D1) and Domain 2 (D2), respectively [106, 108]. It is unclear why Cdc48 has two AAA+ domains when PAN can perform the same function with only one AAA+ domain. Current data suggests that D1 may stabilize the hexameric complex [114, 115] and D2 primarily hydrolyzes ATP to drive polypeptide translocation [108, 115]. However, mutation of the Walker-B glutamate to glutamine in either D1 or D2 is lethal to cells, suggesting that ATP hydrolysis at both ATPase domains is necessary for proper cellular function [116, 117]. This is reminiscent of the hexameric* E. coli* polypeptide translocase ClpA, which also has two tandem AAA+ domains per monomer. For ClpA, polypeptide translocation is cooperatively driven by both AAA+ domains in the absence of the associated ClpP protease but shifts to a D2-driven translocation mode upon association with ClpP [29, 76, 118, 119]. Analogously, Cdc48 may require ATP hydrolysis at both D1 and D2 for nonproteolytic cellular functions but may switch to D2-driven translocation after association with the 20S particle.

## 6. Concluding Comments

Proteins belonging to the AAA+ family of proteins are structurally similar, but functionally diverse due to N- or C-terminal elements appended to the conserved core *α*-*β*-*α* fold. Here we have highlighted features that characterize the overall AAA+ family and discussed specific examples of archaeal AAA+ family members. Despite the different biological functions and subtle differences in structure, it is clear that substrate-translocating AAA+ proteins interact with and process DNA or polypeptide in fundamentally similar ways that involve substrate translocation through an axial channel within their respective hexameric complexes. This appears to be universal for all organisms including bacteria, archaea, eukaryotes, and even viruses. AAA+ proteins of archaeal organisms have revealed many critical structure-function properties thereby providing a framework for understanding more complex eukaryotic counterparts. Because AAA+ proteins are required for critical biological functions, the basic mechanistic features elucidated with archaeal AAA+ proteins will likely continue to advance our understanding of disease states associated with dysfunction in these proteins.
